# Neurodevelopmental Effects of Low-level Prenatal Mercury Exposure From Maternal Fish Consumption in a Mediterranean Cohort: Study Rationale and Design

**DOI:** 10.2188/jea.JE20120030

**Published:** 2013-03-05

**Authors:** Francesca Valent, Milena Horvat, Aikaterini Sofianou-Katsoulis, Zdravko Spiric, Darja Mazej, D’Anna Little, Alexia Prasouli, Marika Mariuz, Giorgio Tamburlini, Sheena Nakou, Fabio Barbone

**Affiliations:** 1 Institute of Hygiene and Epidemiology, Department of Medical and Biological Sciences (DSMB), University of Udine, Udine, Italy; 2 Institute of Hygiene and Clinical Epidemiology, University Hospital of Udine, Udine, Italy; 3 Jozef Stefan Institute, Ljubljana, Slovenia; 4 Institute of Child Health, Athens, Greece; 5 OIKON Ltd, Institute for Applied Ecology, Zagreb, Croatia; 6 Institute for Maternal and Child Health-IRCCS Burlo Garofolo, Trieste, Italy

**Keywords:** cohort study, mercury, polyunsaturated fatty acids, nervous system development, fish

## Abstract

**Background:**

Mercury is a neurotoxic environmental pollutant. However, the literature on the neurodevelopmental effect of low-level prenatal mercury exposure from maternal fish intake is inconsistent. We assessed the association between prenatal mercury exposure and infant neurodevelopment in coastal areas of 4 Mediterranean countries.

**Methods:**

This was a prospective cohort study that planned to enroll approximately 1700 mother–infant pairs. Pregnant women and their newborn children were recruited in selected hospitals of the study areas. Biological samples, including maternal hair and cord blood, were collected from mothers and children, and the concentrations of mercury and other elements were measured. Exposures to lifestyle, environmental, and social factors were assessed through questionnaires. The main outcome was child neurodevelopment at 18 months, as measured by the Bayley Scales of Infant and Toddler Development, Third Edition.

**Conclusions:**

This cohort has a number of strengths. First, mercury concentration was measured in several biological samples, which allows for a better understanding of mercury kinetics and is useful for sensitivity analyses. Therefore, we expect to be able to adjust for the potential confounding effects of lifestyle and social factors and for the effects of other elements that were measured in the biological samples. Finally, this is a multinational study and thus permits assessment of the relation between mercury and child neurodevelopment in different populations.

## INTRODUCTION

Mercury (Hg) is an environmental pollutant that can have neurotoxic effects on the human central nervous system, particularly during fetal development.^1^ Two major incidents, in Minamata^2^ and Iraq,^3^ showed the effects among local populations of acute exposure to high levels of mercury through contaminated food and provided strong evidence for mercury toxicity in the fetus. However, severe mercury poisoning is unusual. Chronic low-level exposure (<10 ppm in maternal hair) is more common and is primarily due to consumption of fish and other seafood. Methylmercury (MeHg), the organic, neurotoxic form of mercury that is produced from inorganic mercury by biomethylation in aquatic sediment, bioaccumulates in fish. The highest concentrations are seen in the muscle of larger, older predator fish.^4^

A number of cross-sectional and prospective cohort studies assessed the neurodevelopmental effects of chronic low and moderate prenatal MeHg exposure from maternal fish consumption.^5^^–^^16^ However, the findings were inconsistent, possibly because of differences in outcomes, biomarkers, confounders, study populations, mercury concentrations, and other contaminants in fish.

Despite those inconsistencies, health agencies in various countries used the data from the largest cohorts to determine the risk of MeHg ingestion to the general population. The World Health Organization (WHO) used data from the Faroe Islands, the Seychelles Islands, and New Zealand to establish a weekly tolerable dietary intake of 1.6 µg/kg (0.23 µg/kg/day) MeHg.^17^ The US Environmental Protection Agency (EPA) also used data from those studies to establish an oral reference dose of 0.1 µg/kg/day.^18^ In addition, the US Food and Drug Administration (FDA) and EPA recommend that women of childbearing age limit their fish intake and that they particularly avoid large predator species, so that, in case of pregnancy, mercury exposures potentially harmful to the fetus would be avoided.^19^^,^^20^ In a cohort of pregnant women in Massachusetts, the reported consumption of fish diminished after dissemination of these recommendations,^21^ limiting the potential exposure to mercury but also the intake of polyunsaturated fatty acids (PUFAs), which are essential for optimal fetal neurodevelopment.^22^ Thus, there is a need for further research so that clear and correct indications regarding the risks and benefits of seafood intake during pregnancy can be provided to women.

Recently, small Italian and Greek studies found that total mercury (THg) concentrations in hair samples from pregnant women and mothers of young children were directly associated with the consumption of local fish and reached levels of approximately 10 ppm or higher.^23^^,^^24^

We designed a prospective cohort to investigate the effects of prenatal low-level mercury exposure from maternal consumption of fish and seafood on child neurodevelopment among residents of the Mediterranean coastal areas of 4 neighboring countries.

## METHODS

This prospective cohort study was set within a 5-year integrated project on the public health impact of long-term, low-level, mixed element exposure in susceptible population strata (PHIME). The project began in 2006 and was completed in 2011. The aim of the PHIME project was to improve the integrated health risk-assessment of long-term, low-level environmental exposure to toxic and essential metals via food. Within this larger framework, the present cohort particularly aimed to assess the association between mercury exposure from food consumption during pregnancy and development of the nervous system (http://phime.oikon.hr/).

### Setting

The cohort includes 4 recruitment areas: (a) the coastal Province of Trieste, Italy, (b) the city of Ljubljana, Slovenia and its surroundings (up to 50 km), (c) the coastal city of Rijeka, Croatia and its county (Primorsko-goranska), and (d) the Greek islands of Lesvos, Chios, Samos, and Leros in the eastern Aegean. The overall number of births in the study areas was estimated to be approximately 7000 per year.

### Recruitment

The pregnant women eligible for recruitment were permanent residents of the study areas for at least 2 years, were at least 18 years of age, and had no absence from the study area for more than 6 weeks during pregnancy, no history of drug abuse, no serious health problems or complications of pregnancy, and no twin gestation. All participants attended 1 of the recruitment centers for routine ultrasound scans or delivery. Recruitment took place at the Burlo Garofolo Children’s Hospital in Trieste, Italy; at the Maternity Hospital of the University Medical Centre of Ljubljana, Slovenia; at the University Hospital of Rijeka, Croatia; and at the general regional hospitals of Mytilini (Lesvos), Chios, Samos, and Leros in Greece.

At recruitment, eligible women were approached for consent after their routine morphologic ultrasound scan between 20 and 22 gestational weeks (Italy), at routine visits between 34 and 38 gestational weeks (Croatia), or during their hospital stay for delivery (Slovenia, Croatia, and Greece). Times for enrollment were chosen according to logistic considerations in each country because there is no evidence that a particular gestational age is optimal for enrollment in this type of study. In fact, gestational age has varied considerably in cohort studies of prenatal mercury exposure and neurodevelopment.^16^^,^^25^^–^^28^ Written informed consent was obtained by study researchers. Participation was explained, all questions were answered, and women who agreed signed the consent form. The original was retained and placed in the participant’s documentation folder. An identification code was assigned to each participant and was used for all materials collected from the same woman and her child.

Due to logistic issues, the timing and amount of data and samples collected were different among the 4 centers. The Table shows the study phases and the data and samples collected in each country. The data collection instruments and sampling methods were the same across the 4 countries, and the analysis of all biological samples was performed in the same laboratories.

**Table.** Protocol summary: study phases, samples, and data collected during each phase in each country of the Northern Adriatic Cohort II and Eastern Aegean Cohort

	Recruitment	Short questionnaire	Maternal hair (first sample)	Maternal blood	Maternal urine	Raven’s test	Long questionnaire	Cord blood	Meconium	Maternal hair (second sample)	Breast milk	Child hair	BSID III	M-CHAT	Supplementary questionnaire
Italy	20–22 weeks EGA	20–22 weeks EGA	20–22 weeks EGA	20–32 weeks EGA	20–32 weeks EGA	20–32 weeks EGA	28 weeks EGA–1 month after delivery	At birth	—	1 month after delivery	1 month after delivery	18 months	18 months	18 months	18 months

Slovenia	At birth	At birth	At birth	—	—	—	1 month after delivery	At birth	At birth	—	1 month after delivery	—	18 months	18 months	18 months

Croatia	34 weeks EGA–birth	34 weeks EGA–birth	34 weeks EGA–birth	34 weeks EGA–birth	34 weeks EGA–birth	At birth	1 month after delivery	At birth	At birth	—	1 month after delivery	—	18 months	18 months	18 months

Greece	At birth	At birth	At birth	—	—	—	3–6 months after delivery	At birth	At birth	—	3–6 months after delivery	—	18 months	18 months	18 months

EGA: estimated gestational age.

### Follow-up

After delivery, ie, some weeks before each follow-up visit up to a child age of 18 months, the women were contacted by mail to remind them of the goals of the research. Then, they were telephoned by a researcher to assess their willingness to participate in the relevant phase and schedule an appointment. Feedback on individual results of previous laboratory exams and tests was provided to families in letters either mailed to their homes or handed to them at subsequent visits.

In Italy, a first birthday card and annual Christmas cards were sent to children to strengthen contact with their families. Follow-up continued in Italy after age 18 months, in the manner described above.

### Criteria for exclusion from follow-up

We excluded from further follow-up any preterm births (<37 weeks of gestational age), babies with congenital malformations or severe perinatal problems, and those with severe health problems that presented in the following months and potentially compromised their neurological development. These exclusion criteria were adopted as the best method for avoiding confounding due to preterm birth, malformations, and health problems, which may be associated with mercury exposure and neurodevelopmental delay. Other methods to control for potential confounding (ie, matching in the design stage, stratification of modeling in the analysis stage) were considered infeasible because of the anticipated low prevalence of such conditions.

### Questionnaires

Three questionnaires were administered to mothers during the study, at different phases. They were designed at the University of Udine, Italy, translated from English into the local languages, and pilot-tested in each country.

A short questionnaire administered by researchers at recruitment was designed to identify any excluding conditions and to provide a quick assessment of demographic information and maternal frequency of consumption of food items (vegetables; milk and milk products; eggs; meat; fresh, frozen, and canned fish; and alcoholic beverages) and smoking status.

A long questionnaire was completed by the mother after delivery (administered by a researcher in Slovenia and Greece) and collected sociodemographic and health status information on the mother and her family, information on the pregnancy and delivery and the health status of the newborn child, a detailed residential and occupational history of the mother, a record of maternal smoking, drinking, and general dietary habits, a detailed food frequency assessment of her consumption of 138 food items adapted from a validated food frequency questionnaire,^29^^–^^31^ and a qualitative section investigating the consumption of over 22 fish species commonly fished or marketed in the study areas. The names of the food items were translated from English into the local languages, using official dictionaries, and then back-translated to verify the quality of translation.

A supplementary questionnaire was administered approximately 18 months after delivery. It assessed changes in residence, maternal marital and occupational status, anthropometric measures and developmental milestones of the child, breastfeeding history, child intake of fish, diseases, and daycare attendance.

### Biological samples

Sampling was done according to a protocol developed by the Jozef Stefan Institute, Ljubljana and by the University Medical Centre, Clinical Institute of Clinical Chemistry and Biochemistry, Ljubljana, using standardized sample containers. Each sample was labeled with the participant’s identification code.

In the second and third trimesters, the following samples were collected from mothers: approximately 1 g of hair cut close to the occipital area, for THg and MeHg determination; one 5-mL tube of serum, for Fe, Mg, Ca, PUFA, and polychlorinated biphenyls (PCBs) determination; one 7-mL tube of plasma (NaH), for THg, MeHg, lead (Pb), cadmium (Cd), arsenic (As), manganese (Mn), zinc (Zn), copper (Cu), and selenium (Se) determination; one 3-mL tube of plasma (K_3_EDTA), for determination of genetic polymorphisms; 50 mL of urine, for THg, Cd, and creatinine determination.

Maternal hair and blood samples were collected by trained ad hoc research personnel in Italy and by hospital personnel in the other countries. Urine was collected by the mothers at home and then brought in to research personnel. In Greece it was collected in the hospital at the time of delivery.

At delivery, the following samples were collected: one 5-mL tube of serum from mixed umbilical cord blood, for Fe, Mg, Ca, and PUFA determination; one 7-mL tube of plasma (NaH) from mixed umbilical cord blood (with NaH), for THg, MeHg, Pb, Cd, As, Mn, Zn, Cu, and Se determination; 20 mL of child urine (optional), for THg and creatinine determination; 3 to 5 cm of cord tissue (optional), for THg, MeHg, Pb, Se, and PCBs determination; 5 to 10 g of meconium (optional), for THg, MeHg, Pb, Se, and PCBs determination.

In Italy, umbilical cord blood or cord tissue samples were collected at delivery by trained research personnel who were on call at all times and were alerted by Burlo Garofolo Hospital midwives when women participating in the study started labor. In the other countries, cord blood and infant urine or meconium were collected by the personnel in the Obstetrics and Gynecology Divisions who assisted the mothers during delivery.

At approximately 1 month after delivery, the following samples were collected: approximately 50 mL of 24-hour breast milk, for THg, Cd, Pb, Se, Zn, PUFA, and PCBs determination; approximately 1 g of maternal hair close to the occipital area, for THg and MeHg determination. At 18 months of age, a lock of child hair was collected for THg determination.

In most cases, breast milk from lactating women, and child hair, were collected by trained research personnel at the participants’ homes; in a small number of cases, mothers preferred to hand in the samples to research staff at the study hospital.

Hair samples were stored in transparent mercury-free plastic bags in a dark and uncontaminated place and were periodically sent to Jozef Stefan Institute of Ljubljana for analysis. Aliquots of blood, urine, milk, and cord tissue samples were stored in freezers (below −24°C) and then sent or transported in a frozen state (dry ice) to the following laboratories for analysis: the Jozef Stefan Institute, for determination of THg and MeHg in all types of samples, and for multielemental analysis of whole blood, milk, and urine samples; the University Clinical Centre of Ljubljana, for measurement of Zn and Se in plasma samples and creatinine in urine samples; Lund University, for genotyping; and University of Ulster, for measurement of PUFAs.

### Outcome assessment

Child neurodevelopment was assessed at 18 (range, 16–20) months of age by using the Bayley Scales of Infant and Toddler Development, Third Edition, Screening Test (BSID-III).^32^ All 5 scales (cognitive, language, motor, social emotional, and adaptive behavior) were measured in all countries except Greece, where the adaptive behavior scale was not measured. Both the scaled and composite scores were calculated.

The tests were conducted in each country by trained pediatricians or psychologists (and by trained psychology students in Slovenia) in the study hospitals. In countries where more than 1 tester conducted the BSID, inter-rater reliability was estimated. In Italy, the BSID is administered again at 40 months of age.

### Other tests

In Italy (at 20–32 gestational weeks) and Croatia (during the perinatal period), mothers in the study took the Raven’s Progressive Matrices Test.^33^ At 18 months, children were also screened for autism by using the Modified Checklist For Autism in Toddlers (M-CHAT),^34^ which was performed by the same psychologists and pediatricians who administered the BSID, and on the same day.

In Italy, the AIRE (a method for evaluating family environment, designed by Capotorti^35^ and based on the HOME model^36^) is conducted at the child’s home when he or she is between age 18 and 40 months. Through a combined process (interview plus observation), 4 specific fields (subscales) are explored: communication between children and parents (***A****ffetto*), promotion of autonomy (***I****ncoraggiamento*), **R**espect for children and implementation of rules, and **E**motional atmosphere.

### Ethics

The research protocol was approved by the Ethics Committees of the University of Udine, the Burlo Garofolo Children’s Hospital of Trieste, the Clinical Center of Ljubljana, the Clinical Center of Rijeka, and the Institute of Child Health of Athens. All aspects of the study, including ethics, were monitored annually by the European Commission.

### Data entry and quality control

In each country, all data collected on paper forms were entered into electronic databases prepared by the University of Udine. The electronic files were then sent to the University of Udine for quality control and statistical analysis.

### Statistical analyses

Mercury exposure is assessed in all subjects who provided at least 1 biological sample, at any visit, for laboratory analysis of mercury. Women enrolled in the cohort who subsequently withdraw or were lost to or excluded from follow-up may be included in the descriptive analyses of cohort exposures at baseline. However, only children who underwent both 1 or more measurements of mercury exposure and neurological testing at 18 months are included in the statistical analyses of the association between exposure and outcome.

Analyses are conducted for the whole cohort (Italy + Slovenia + Croatia + Greece), using a subset of common variables; larger sets of variables are used in country-specific analyses. Multilevel models will be used for the analyses of whole countries, to account for intracountry clustering. Such models are useful in separately estimating the predictive effects of an individual exposure and its group-level (in this case, country) mean, which are sometimes referred to as the “direct” and “contextual” effects of the predictor.

The variables considered for the analyses can be grouped into the following categories: maternal demographic variables (age at delivery, country of birth, marital status, height, weight before pregnancy, weight at delivery, body mass index, occupation); maternal intelligence (Raven score); housing conditions and socioeconomic indicators (house property and size, number of adult and child cohabitants, number of cars in family, maternal and paternal educational level, occupation of father, home environment at follow-up, ie, AIRE); pregnancy history (use of medications—type and indications, intake of folic acid integrators, dental visits, new or replaced dental fillings, smoking habit and number of cigarettes smoked, exposure to passive smoking at home and at work, dietary habits, including consumption of fruits with peel, frequency of consumption of prespecified food servings, alcohol consumption, frequency of consumption of fish species, pre-eminent fresh fish suppliers in winter and summer and locations, environmental exposures such as house location and sources of environmental pollution); delivery and characteristics of child (gestational age, type of delivery, sex of child, birth weight, length, child hospital admissions in first month after birth and cause); child postnatal exposures (hospital admissions and cause, duration of breastfeeding, daycare attendance, fish intake); other variables from questionnaires (season of Bayley test administration, time of day of Bayley test administration, child age at Bayley testing); and biochemical variables (PUFAs, creatinine, and multielemental concentrations in different biological samples).

Analyses of the association between prenatal mercury concentration in each biological sample (with or without transformation) and child neurodevelopment is conducted through multivariate linear regression, adjusted for the effect of potentially confounding variables. Separate models are built for each dependent variable (Bayley score).

Because cord blood was collected at delivery in all 4 countries, mercury concentration in cord blood is considered the main exposure of interest. Mercury concentration in hair was measured at different times in different countries. Mercury concentration in hair varies during pregnancy, although little is known of the magnitude of such variations. In addition, at low exposures, external contamination of hair may also contribute to observed variability. Nonetheless, we used mercury concentration in hair as an approximate measure of mercury exposure during pregnancy because hair was easily collected from nearly all participants, because the findings could be compared with those of other studies, and because it is a good measure of long-term average exposure.^33^ We therefore expect to find a correlation between the average frequency of consumption throughout pregnancy of food items known to be potentially contaminated with mercury (eg, fish and seafood), as reported by mothers at the end of pregnancy or soon after delivery, and the average concentration of mercury in hair, as estimated from hair samples.

### Power calculations

We estimated the sample size required to obtain a statistically significant result in the case of a moderate association with a relatively low level of exposure. Assuming that the prevalence of a measurable neurodevelopmental delay is 10% among children whose mother’s hair has a THg level of ≥4.0 µg/g (n_1_) and 5% among children whose mother’s hair has a THg level of <4.0 µg/g (n_2_), and that the n_2_/n_1_ ratio = 1, α = 0.05, and β = 0.10, the sample size needed to estimate a risk ratio of 2.0 is n_1_ = 621, n_2_ = 621. Given the personnel and time available, we estimated that we would be able to enroll approximately 1700 mother–child pairs during the study period (750 from Italy, 350 from Slovenia, 200 from Croatia, and 400 from Greece).

### Limitations

The BSID is rather time consuming (approximate duration of each 18-month visit, 2 hours), and appointments must be carefully scheduled. This is particularly challenging in Greece, where study personnel must travel to many islands.

The inclusion of 4 countries in the cohort complicated the study design because the protocol had to be slightly adapted to suit the different cultural contexts and logistic needs. For example, recruitment was initially planned to occur during pregnancy, so that mercury concentrations in biological samples would reflect fetal exposures at different gestational ages. However, this was unfeasible in countries other than Italy. Similarly, collection of some information, such as maternal intelligence through the Raven’s test, was easier in some contexts than in others. Differences in the amount of information available in each country will limit the set of variables to be included in the overall analyses. More detailed analyses will thus be country-specific.

### Strengths

Despite the above-mentioned practical issues, the availability of data from a multinational cohort will certainly add value to our research and allow for greater variability in the exposures under study and hence a better assessment of dose-response relationships. It will also increase understanding of the effects of prenatal mercury exposure on neurodevelopment in populations that, although similar in many aspects, are exposed to different genetic, cultural, and environmental factors. Multilevel analyses will be extremely useful in accounting for differences in the timing of collection of some biological samples and questionnaires by country, as well as for intracountry clustering due to other factors (ie, cultural aspects). In fact, people living in the same country tend to be more similar to each other than people randomly sampled from the entire Mediterranean and Eastern Aegean area. In our cohort, multilevel modeling will allow us to disentangle individual (eg, mercury exposure) and group (eg, country-specific factors) effects on BSID-III scores. This cohort will also allow us to explore gene–environment interactions in the toxicokinetics of mercury.

In this Mediterranean cohort we measured mercury concentration in several biological samples. This is a great advantage over previous studies^5^ and will permit us to better understand mercury kinetics, and assess the sensitivity of estimated associations between mercury and neurodevelopment, using concentrations in different samples. Mercury concentration in hair is a robust approximate measure of mercury exposure during pregnancy^37^^,^^38^ and will allow comparisons with other studies, although we are fully aware that, at low exposures, external contamination of hair may also contribute to observed variability. For this reason we also sampled cord blood, which is a measure of uterine methylmercury exposure. Cord blood was taken from all 4 countries in the Mediterranean cohort, so comparability among the 4 countries is best when cord blood is taken as a marker of prenatal mercury exposure.

In addition to THg and MeHg, we measured concentrations of a number of other neurotoxic and beneficial trace elements, as well as nutrients essential to the developing nervous system. This will enable us to control for their potential confounding effects on the association between mercury and neurodevelopment, thereby avoiding the imprecision and information biases that can affect estimates of nutrients and contaminants that are based solely on questionnaires.^39^
